# The prevention of anaphylactoid reactions to iodinated radiological contrast media: a systematic review

**DOI:** 10.1186/1471-2342-6-2

**Published:** 2006-04-27

**Authors:** Anthony Delaney, Andrew Carter, Malcolm Fisher

**Affiliations:** 1 Intensive Care Unit, Royal North Shore Hospital, Pacific Highway, St. Leonards, NSW, 2065, Australia; 2 Northern Clinical School, University of Sydney, St. Leonards, NSW, 2065, Australia; 3 Department of Radiology, Concord Hospital, Hospital Rd, Concord, NSW, 2139, Australia

## Abstract

**Background:**

Anaphylactoid reactions to iodinated contrast media are relatively common and potentially life threatening. Opinion is divided as to the utility of medications for preventing these reactions. We performed a systematic review to assess regimes for the prevention of anaphylactoid reactions to iodinated contrast media.

**Methods:**

Searches for studies were conducted in the Medline, EMBASE, CINAHL and CENTRAL databases. Bibliographies of included studies and review articles were examined and experts were contacted. Randomised clinical trials that examined agents given prior to iodinated contrast material for the prevention of anaphylactoid reactions were included in the review. The validity of the included studies was examined using a component approach.

**Results:**

Six studies met the inclusion criteria, but only one of these fulfilled all of the validity criteria. There were four studies that examined the use of H1 antihistamines, each was used to prevent anaphylactoid reactions to ionic contrast. The random effects pooled relative risk demonstrated a significant reduction in the overall rate of anaphylactoid reactions (RR = 0.4, 95% CI 0.18-0.9, p = 0.027). There were insufficient studies to produce a pooled statistic for the use of corticosteroids, however regimes of steroids (methylprednisolone 32 mg) given at least six hours and again two hours prior to the administration of contrast suggested a reduction in the incidence of anaphylactoid reactions.

**Conclusion:**

In conclusion, there are few high quality randomised clinical trials that have addressed the question of the optimal methods to prevent allergic type reactions to iodinated radiological contrast media. Allowing for these limitations, the results suggest that H1 antihistamines given immediately prior to the administration of ionic contrast may be useful in preventing reactions to ionic contrast and are suggestive of a protective effect of corticosteroids when given in two doses at least six hours prior and again two hours prior to the administration of contrast, both ionic and non-ionic. These agents should be considered for use in patients who are at high risk of an anaphylactoid reaction to contrast media and for who prophylactic therapy is considered necessary. Further research is needed before definitive recommendations can be made.

## Background

Advances in diagnostic and interventional radiology have meant that the number of radiological procedures that require use of iodinated contrast media have increased in recent years. The use of iodinated contrast media is associated with a risk of adverse reactions, including serious allergic type reactions which may be life threatening. Reactions to iodinated contrast range from minor flushing, to severe life threatening anaphylactoid or even fatal reactions. While the precise mechanism is not well understood, IgE mediated, complement mediated and cell mediated reactions may play a role [1,2]. The incidence of adverse reactions to iodinated contrast has been estimated to be 12.66% for ionic and 3.13% for non-ionic contrast. Severe reactions which were potentially life-threatening can occur in 0.04% to 0.22% of cases [3]. Even with a small incidence such as this, because there are an estimated 50 million procedures involving iodinated contrast media performed worldwide each year, there are a large number of patients at risk from potentially life-threatening reactions[4].

A number of agents have been suggested as being useful for the prevention of anaphylactoid reactions to iodinated contrast material, including corticosteroids and H1 and H2 anti-histamines. There is no current consensus on the optimal therapy that should be used as preventative agents, or even if medication should be used now that the use of non-ionic contrast is almost universal[5,6]. Even with the widespread use of non-ionic contrast, many radiologists continue to use prophylactic medication to prevent allergic type reactions[7]. Previous guidelines have used consensus methods rather than evidence-based methods to make recommendations [6]. While there has been much recent interest in this area, recent reviews have not focused specifically on the use of medications in the prevention of anaphylactoid reactions and have not used systematic methods to review the literature [1,4]. Systematic reviews of randomised controlled trials are preferred to opinion-based guidelines to guide clinical practice, and may lead to less biased and more reliable recommendations [8,9]. Systematically searching for and critically appraising the available primary studies is also important to allow clinicians the opportunity to evaluate the strength of the evidence that forms the basis of current practice, and may point out opportunities for researchers seeking to improve that evidence.

No previous study has applied systematic methods to reviewing the literature regarding the prevention of anaphylactoid reactions to iodinated contrast media. The aim of this study was to systematically review the literature regarding the use of medication in the prevention of anaphylactoid reactions to contrast material and to determine which medication or medications when given prior to the administration of the contrast media, are associated with a reduced incidence of these reactions.

## Methods

### Search strategy

The primary search for relevant studies was conducted in a number of electronic databases. The MEDLINE database was searched using the Pubmed interface, and this was supplemented with searches of MEDLINE, EMBASE, CINAHL and the Cochrane central register of controlled trials, using the OVID interface. Search terms were individualised for each database and included MESH headings and text words; (anaphylaxis, anaphylactic, anaphylactoid, hypersensitivity, allergic reaction and adverse reaction), combined with ("prevention and control" or prevention) and (contrast media or radiological contrast). The bibliographies of the included articles and review articles were examined and attempts were made to contact experts in the field to identify any otherwise unidentified or unpublished studies. The final search was conducted up until the June 9^th^, 2005. There were no language restrictions placed on the search.

### Inclusion of studies

The titles and abstracts of all reports were reviewed by one author to identify potentially relevant articles. The full text reports of potentially relevant studies were then retrieved and reviewed for consideration for inclusion in the study. Two authors (AD and AC) independently applied the pre-determined inclusion criteria to determine eligibility of the article for inclusion in the review. Disagreements were resolved by discussion. To be eligible for inclusion the report had to describe a study:

1. of a medication or medications given prior to the administration of contrast for a x-ray examination

2. where the x-ray contrast was given as an injection

3. where the main outcome measure was the rate of allergic reactions

4. that was a prospective randomised clinical trial

5. that was reported in English [10]

### Validity assessment

The validity of the included studies was assessed using a component approach, with each study assessed for the adequacy of randomisation and allocation concealment, the blinding of outcome assessment and the presentation of an intention-to-treat analysis and whether the definitions of what constituted an allergic reaction were prospectively defined. Studies that used a method of randomisation that did not maintain allocation concealment, such as alternate days or medical record numbers (i.e. were pseudo-randomised) were recorded as not maintaining allocation concealment. The validity assessments were conducted by two reviewers (AD and AC), independently, with disagreements resolved by discussion.

### Data extraction

Data were extracted independently, in duplicate by two authors (AD and AC) onto a specifically designed data collection form. Disputes were resolved by discussion. Data were extracted from the reports regarding the population under study, the type of contrast used, the prophylactic regime used and the rates of anaphylactoid reactions. It was decided a-priori to assess the reactions in two groups where possible, all anaphylactoid reactions and those which were potentially life threatening such as bronchospasm, hypotension and airway oedema.

### Data synthesis and quantitative analysis

Prophylactic regimes were grouped according to the pharmacological action of the agents under consideration. Statistical pooling of results was only considered when there were three or more studies examining the same prophylactic agent. Statistical heterogeneity was assessed using the *I*^2 ^statistic [11] and the χ^2 ^statistic. When heterogeneity was detected the random effects model of DerSimonian and Laird was used to pool the results. The potential for publication bias was assessed using the methods described by Egger [12]. All statistical calculations were performed using STATA 8.2 (Statacorp, College Station, Texas).

## Results

**Figure 1:**
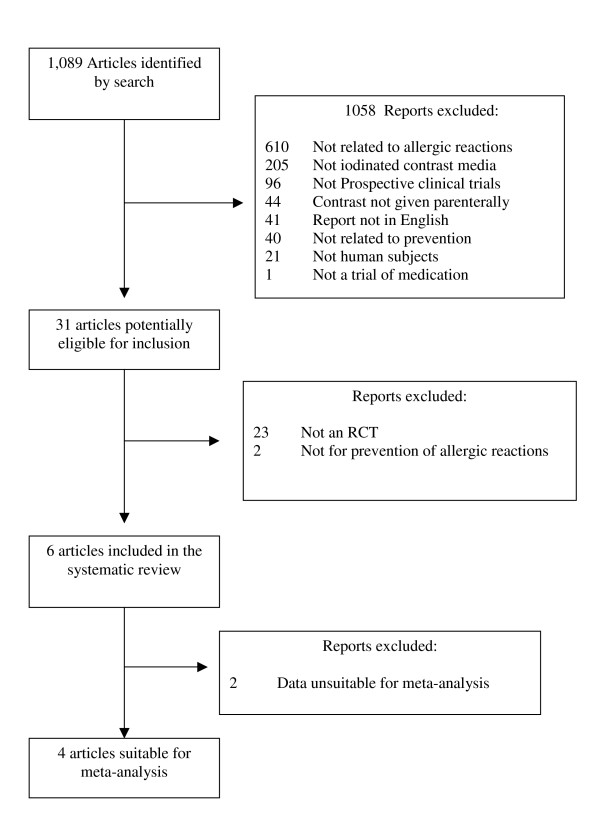
Flow chart showing the flow of included studies and the reasons for exclusion of studies.

A total of 1,099 articles were identified in the initial search. Of these 1068 were deemed ineligible on review of the titles and abstracts and 31 were retrieved for full review. Figure 1 shows the flow of studies and the reasons for exclusion. Six studies met the inclusion criteria. The details of the studies including the prophylactic regimes tested are shown in Table 1. Table 2 shows the results of the validity assessments for each of the included studies. Only one study clearly met all of the predefined validity criteria.

### Anti-Histamines

**Figure 2:**
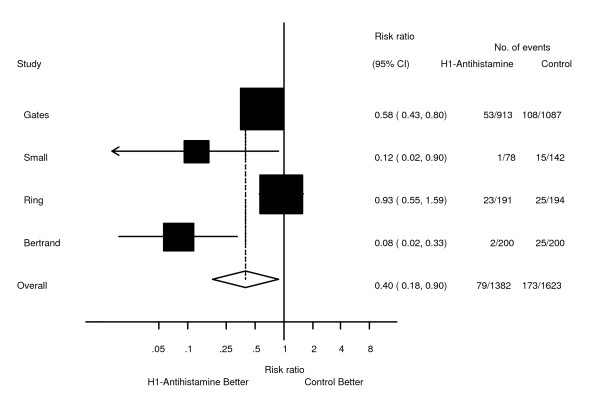
Forest plot showing the effect of prophylactic H1 antihistamines on the incidence of anaphylactoid reactions to iodinated contrast media.

Four studies examined the use of H1-antihistamines [13-16]. No study met all of the pre-defined validity criteria. One study [15] included three treatment groups, so for the purpose of this analysis only the group that received clemastine was considered. There was significant clinical heterogeneity as all examined different antihistamine regimes and the contrast agents were all different. Not surprisingly there was evidence of significant statistical heterogeneity amongst the included studies, with the χ^2 ^test p = 0.003 and the *I*^2 ^= 76.7%. The Egger statistic revealed no evidence for significant publication bias (bias = -2.19, p = 0.32), and inspection of the funnel plot revealed no significant asymmetry. Due to the presence of significant statistical heterogeneity the results from these four studies were pooled using a random effects model (Figure 2), This showed a significant reduction in the risk of allergic reactions with the estimate of the relative risk 0.40 (0.18-0.9, p = 0.027). Separate examination of the reduction in rates of severe events was not possible, as these events were not reported in these trials.

### Combination of H1 and H2 antihistamines

One study [15] included an experimental group treated with a combination of clemastine and cimetidine. They found an incidence of side effects (excluding the subjective sensation of heat) of 6.1% compared to 12.9% in a placebo group. This reduction was reported as statistically significant. Again, there was no reporting in the reduction of the rates of severe events.

### Corticosteroids

Three studies examined the effect of corticosteroids [15,17,18]. There were two separate regimes tested. As only two studies examined each regime, statistical pooling of the results was not possible. Corticosteroids given immediately prior to the administration of contrast was not associated with a significant reduction in the incidence of anaphylactoid reactions. One study recorded an incidence of adverse reactions of 10.1% in the group given prednisolone 250 mg five minutes prior to the infusion of contrast compared to 12.9% in the group given saline [15], and the other study recorded an incidence of 9.4% in the group given a single dose of 32 mg of oral methylprednisolone 2 hours prior to the administration of contrast, compared to 9.9% in the comparable placebo group [17].

However when the steroids were given in two doses, at least six hours prior to, and again two hours prior to the administration of contrast, the incidence of anaphylactoid reactions was reduced. The incidence of reactions was significantly reduced in patients receiving the methyl prednisolone 32 mg as a two-dose regime to 6.4% from 9.0% in the placebo group (p < 0.0001) [17]. The rate of severe reactions in this study was reduced from 0.75 to 0.2% (p = 0.04). Ionic contrast used in this study. The effect of steroids on patients receiving non-ionic contrast was examined in a latter study [18]. This study was the only one to fulfil all of the validity criteria. The rate of overall reactions to non-ionic contrast in this study was reduced from 5% in the placebo group to 2% in the group treated with a two-dose regime of methylprednisolone. There was a non-statistically significant reduction in the rate of severe reactions from 0.87% to 0.17% (p = 0.11).

## Discussion

With the increasing numbers of radiological procedures that require the use of intravenous contrast, the prevention of anaphylactoid reactions is likely to remain an important issue both for radiologists performing the procedures and the physicians referring patients for these investigations. This systematic review found that there were relatively few studies that examined treatments to prevent anaphylactoid reactions to intravenous contrast, and that the studies that are available are generally not of a very high quality. Allowing for these limitations, the results suggest that H1 antihistamines given immediately prior to the administration of ionic contrast may be useful in preventing reactions to ionic contrast and are suggestive of a protective effect of corticosteroids when given in two doses at least six hours prior and again two hours prior to the administration of contrast, both ionic and non-ionic. These agents should be considered for use in patients who are at high risk of an anaphylactoid reaction to contrast media and for who prophylactic therapy is considered necessary.

As the overall incidence of anaphylactoid reactions to contrast media is low, the use of prophylactic medication is likely to be restricted to those most at risk, such as patients with previous reactions to contrast and those with asthma [19]. The protective effect of H1 antihistamines and corticosteroids in high-risk patients has been demonstrated in non-randomised studies. The use of prednisone 50 mg given 13, 7 and 1 hour and diphenhydramine 50 mg 1 hour prior to the administration of non-ionic contrast in 120 patients who had previous reactions to contrast was associated with only one minor reaction [20]. For emergency procedures, the use of hydrocortisone in a doses of 100–250 mg given intravenously as soon as the need for the examination was established and then every 4 hours until the contrast examination was completed, combined with diphenhydramine 50 mg given immediately prior to the examination was associated with no allergic reactions in a series of nine patients with previous allergic reactions to contrast [21]. There are however reports of patients who have had repeat reactions in spite of premedication [22-24].

The incidence of allergic reactions is also dependent on the type of contrast agent used. The use of non-ionic contrast has been associated with a lower risk of allergic reactions[19]. There is still a small but significant risk of serious allergic reactions when non-ionic agents are used [3,25]. As the study by Lasser[18] was the only study to examine the effect of prophylactic treatment for patients receiving non-ionic contrast, the results of this review would suggest, without being definitive, that two doses of corticosteroids be used as prophylaxis, at least for patients at high risk of reactions. This is the approach being already used by some clinicians[7]. Corticosteroids would not however be expected to prevent all reactions, especially those mediated by chemotoxic reactions.

There are a number of limitations to the results that can be drawn from this study. Firstly, the quality of the included studies was less than optimal. The strength of a meta-analysis is reliant on the quality of the included studies. When the primary studies are of a poor quality, no statistical manipulation can make up for the inherent potential bias. Secondly, there was significant heterogeneity with regards to the prophylactic agents used with each study using a different agent, timing and route of administration, the contrast agent used and the populations under investigation. Thirdly, while combinations of medications are often recommended [6], only one of the studies identified in this review examined the effect of combinations of medications. Fourthly, most of the studies included in this review did not specifically examine the effect of the prophylactic regimes in high-risk patients, or the effects of these therapies for patients receiving non-ionic contrast, the specific population for whom prophylactic therapy is generally aimed [6]. These factors limit the conclusions that can be drawn from this study.

One of the most important findings of this review is that there is little current high-quality evidence that clinicians could rely on to guide their practice. By identifying the gaps in the evidence using systematic methods, it is hoped that this review may form the foundation for further studies to improve knowledge in this area. While randomised clinical trials are often viewed as the gold standard for evidence for preventative or therapeutic interventions, the difficulty in performing large randomised control trials to examine this issue may be prohibitive. It has been argued that well conducted observational studies can produce estimates of treatment effect that reflect the results produced by randomised clinical trials [26,27]. Large properly designed cohort or case-control studies would be more feasible and may be the appropriate study designs to shed further light on these issues. Future studies should concentrate on high-risk patients receiving non-ionic contrast. Possible research designs could include large, multi-national co-operative purpose-designed prospective databases. The need for such reporting has been recently highlighted[28]. Such case control or cohort studies could help in deciding whether further prospective randomised double blind placebo controlled trials are necessary and feasible.

## Conclusion

In conclusion, there are few high quality randomised clinical trials that have addressed the question of the optimal methods to prevent allergic type reactions to iodinated radiological contrast media. There is only one underpowered study that examines preventative regimes for ionic contrast agents. This systematic review found that the use of H1 antihistamines and corticosteroids is supported by the available trials that have examined treatments to prevent anaphylactoid reactions to radiological contrast media. However, further research into the appropriate populations to offer this treatment to and the optimal combination of agents to use needs to be considered before recommendations could be made with confidence.

## Competing interests

The author(s) declare that they have no competing interests.

## Authors' contributions

All authors contributed substantially to the production of the manuscript. MF and AD conceived the study, all authors developed the study protocol. The search, study selection and data abstraction were conducted by AD and AC. Analysis was conducted by AD. All authors contributed to the drafting of the manuscript and have approved the content of the manuscript.

## Pre-publication history

The pre-publication history for this paper can be accessed here:



## Figures and Tables

**Table 1:** Characteristics of the included studies

**Study**	**Population**	**Risk Factors for Allergic Reactions**	**Prophylactic regime**	**Contrast agent**	**Reaction**
Gates 1972 [13]	2000 adults undergoing IVP	History of allergic reactions taken, patients with positive history included	Diphenhydramine 10 mg ivi 1 minute prior to contrast	30–60 ml Renograffin 60 (Ionic)	Not defined
Small 1982 [14]	220 adults undergoing IVP	Patients taking anti-histamines excluded	Chlorpheniramine 10 mg sci 15 minutes prior to contrast	Not recorded	Hives, generalised pruritis
Bertrand 1992 [16]	400 adults undergoing IVP, CT or lower limb venography	Patients with history of allergy, atopy, drug sensitivity or previous reactions to contrast excluded	Hydroxyzine 100 mg po 12 hours prior to contrast	Meglumine ioxaglate (Ionic)	Nausea, vomiting, hives, urticaria, flushing, facial oedema, wheezing, dyspnoea, laryngeal oedema, hypotension, death
Ring 1985 [15]	800 adults undergoing IVP	Patients with previous life-threatening contrast reaction excluded	1. Prednisolone 250 mg ivi, 5 minutes prior to contrast 2. Clemastin 0.03 mg/kg ivi, 5 minutes prior to contrast 3. Clemastin 0.03 mg/kg ivi + cimetidine 5 mg/kg vi, 5 minutes prior to contrast	100 ml meglumine amidotrizoate (ionic)	Flush, urticaria, angioedema, respiratory symptoms, vomiting, tachycardia, hypotension
Lasser 1987 [17]	6763 adults undergoing IVP or contrast enhanced CT	Patients with previous severe reaction excluded. Patients with general allergies, drug allergies and previous non life threatening reactions were included	1. Methylprednisolone 32 mg po two hours prior to contrast 2. Methylprednisolone 32 mg o the night before + two hours prior to contrast	Various ionic contrast agents	Grade 1: emesis, nausea, sneezing, vertigo Grade II: Hives, fever, hills, Grade III: Shock, bronchospasm, laryngospasm, laryngeal oedema, loss of consciousness, angioedema
Lasser 1994 [18]	1155 adults undergoing IVP or contrast enhanced CT	Patients with allergies including previous reactions to contrast were included	Methylprednisolone 32 mg po the night before and two hours prior to the contrast	Iohexol, Ioversol (non-ionic)	As per Lasser 1987

**Table 2:** Methodological quality assessment of included studies

**Study**	**Allocation concealment**	**Blinding**	**Intention to treat analysis**	**Predefined outcomes**
Gates 1972 [13]	No	No	Unclear	No
Small 1982 [14]	Unclear	Unclear	Unclear	No
Bertrand 1992 [16]	Unclear	"Double-blind"	Yes	Yes
Ring 1985 [15]	Unclear	"Single"	No	Unclear
Lasser 1987 [17]	Yes	Yes	Unclear	Yes
Lasser 1994 [18]	Yes	Yes	Yes	Yes
